# Meteorological factors on the incidence of MP and RSV pneumonia in children

**DOI:** 10.1371/journal.pone.0173409

**Published:** 2017-03-10

**Authors:** Dan-dan Tian, Rong Jiang, Xue-jun Chen, Qing Ye

**Affiliations:** Clinical Laboratory, The Children´s Hospital of Zhejiang University School of Medicine, Hangzhou, China; Kliniken der Stadt Köln gGmbH, GERMANY

## Abstract

**Background:**

Pneumonia is common in children and mostly caused by many pathogens. The aim of this study was to investigate whether the incidence of pediatric mycoplasma pneumoniae (MP) pneumonia and respiratory syncytial virus (RSV) pneumonia was associated with meteorological factors in Hangzhou, China.

**Methods:**

A total of 36500 pneumonia patients were recruited to participate in the study. Nasopharyngeal swabs were collected for the detection of MP and RSV using real-time polymerase chain reaction (RT-PCR) and direct immunofluorescence (DIF) assays, respectively. We used a distributed lag non-linear model (DLNM) to evaluate the correlations between the MP/RSV incidence and meteorological factors.

**Results:**

The detection rates of MP and RSV were 18.4% and 10.4%, respectively. There was a positive correlation between temperature and the MP infection rate, but RSV infection rate was negatively associated with temperature. Moreover, the impact of temperature on infection with RSV presented evident lag and cumulative effects. There was also an evident lag effect of temperature on the infection rate of MP; however, there was no evident cumulative effect.

**Conclusions:**

In this study, the results showed meteorological factors play an important role in the incidence of these two pathogens. All these results can provide the laboratory basis for the early diagnosis and treatment of pneumonia in children.

## Background

Pneumonia is a major cause of paediatric infectious disease mortality worldwide [1–4]. According to the national vital statistics report, one million children die of pneumonia annually, and pneumonia was the sixth most prevalent cause of death in children aged one to four years old in the United States in 2009 [5]. Pneumonia is the leading cause of in-patient paediatric mortality in China, and it constitutes a serious threat to children’s health [6–8]. Pneumonia can be caused by many pathogens, so it is important to understand the possible causes of pneumonia, and which of these aetiologies are most likely, so that effective therapeutic measures can be taken. Hangzhou is the capital of Zhejiang province in China and is a densely populated city with a resident population expected to reach 900 million by the end of 2015. Hangzhou is located in southern China, along the Yangtze River delta, and has obvious seasonal variation throughout the year. Our long-term clinical observation suggested that children had seasonal epidemics of pneumonia that we hypothesized might be associated with local climate factors. Thus, this study randomly selected children with pneumonia for mycoplasma pneumonia and *r*espiratory syncytial virus testing in Hangzhou between January and December, 2015. Meanwhile, we collected the local meteorological data to investigate whether the infection rates of these two pathogens were associated with the meteorological factors in Hangzhou. We hope that this information will provide a basis for early prevention, treatment and avoidance of unnecessary antibiotic use in children with pneumonia in Hangzhou.

## Materials and methods

### Subject information

For this study, we recruited pneumonia patients from the department of paediatrics in Hangzhou and surrounding areas between January 2015 and December 2015 and collected 100 nasopharyngeal swab specimens from pneumonia patients at the Paediatric Outpatient Department in Hangzhou per day. The diagnosis of pneumonia followed World Health Organization Criteria (1994). All participants showed symptoms and signs of pneumonia, including fever within the last three days(body temperature >37°C); acute respiratory infection symptoms, such as cough and wheezing; and radiographic evidence, such as an increase in pulmonary texture or mottled shadows. All performed procedures were in accordance with the ethical standards of the institutional and national research committee and with the 1964 Helsinki declaration and its later amendments. Verbal informed consent was obtained from the parents or legal guardian of individual participants included in the study (all children under 16 years). Study procedures were approved by Zhejiang university ethics committee.

### Methods for the detection of RSV and MP

In this study, we used the D3 Ultra DFA Respiratory Virus Screening& ID Kit (Diagnostic Hybrids, Inc, Athens, USA) to detect RSV according to the manufacturer’s instruction. A mycoplasma pneumoniae PCR kit (Dana Gen Co. Ltd. Guangzhou, China) was used for mycoplasma pneumonia DNA extraction and detection. Amplification and data analysis were performed with an Applied Bio-systems 7500 real-time PCR system (Applied Bio-systems, Inc. CA, USA) under the following conditions: 93°C for 2 minutes and 40 cycles of 93°C for 45 seconds and 55°C for 60 seconds.

### Meteorological data

Meteorological data for Hangzhou were collected from the website of the China meteorological administration (http://www.cma.gov.cn/). Meteorological data collected included daily maximum temperature, minimum temperature, average temperature, temperature variance, relative humidity and daily rainfall. Statistical methods were employed to assess the effects of daily meteorological data on the risk of MP/RSV infection in children.

### Statistical analysis

The distributed lag non-linear model is a time sequence model based on generalized linear models and generalized additive models, which is advantageous for the analysis of lag and cumulative effects in non-linear processes [9]. Consequently, this model was adopted for fitting the data. The daily MP/RSV infection rate was used as the dependent variable, and a cross-basis matrix was created for temperature and lag time. Confounding variables, including holidays, relative humidity, day of the week and long-term trends, were controlled for in our analysis of the relationship between the temperature and RSV and MP infection rates. The model was as follows: Loge [Y] = crossbasis1+ ns (x1, df) + β1x2 + γ1x3 + γ2x4 + δ; in which Y is the infection rate for MP/RSV on observation date t, crossbasis1 is the cross-basis matrix for daily temperature and maximum lag days obtained using the DLNM software package, “ns” is the natural cubic spline function, “df” is the degrees of freedom, and x1 is the time sequence variable that controls for the long-term trend. In this model, X2 is the relative humidity at day t, and β1 is its coefficient. X3 is the dummy variable for day of week, and γ1 is its coefficient. X4 is the variable designating whether “t” is a holiday, coded according to the holiday arrangements on the Chinese government website, including legal holidays such as the Spring Festival, National Day, and Tomb-sweeping Day, and γ2 was the coefficient. “δ” is the constant of the model. Data manipulation and statistical analyses were performed using SPSS18.0 statistical software and R statistical environment 3.2.3 (DLNM 2.1.3 package).

## Results

### The age distribution of MP/RSV pneumonia children

**Fig 1:**
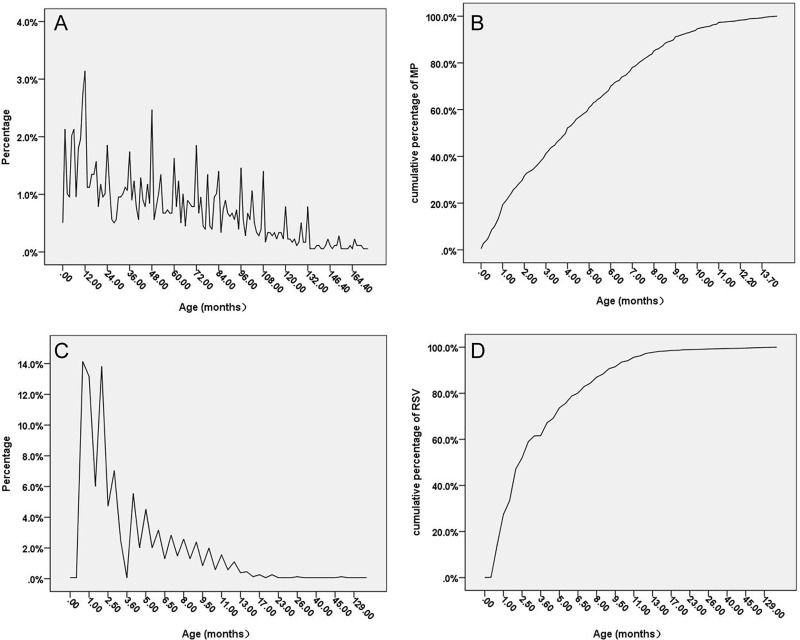
Infection rate for MP/RSV among paediatric pneumonia patients in different age. A: Positive rate for MP by age; B: Cumulative frequency of paediatric MP pneumonia by age; C: Positive rate for RSV by age; D: Cumulative frequency of paediatric RSV pneumonia by age.

The age distributions among children with MP/RSV pneumonia are shown in Fig 1. Of the 36500 paediatric pneumonia cases, 6716 patients (18.4%) were MP positive. In the MP-positive group, the ratio of males to females was 1.3:1. RSV infection was detected in 3796 patients (10.4% of total paediatric pneumonia cases), and the ratio of males to females was 1.8:1in this group (Table 1). MP infection was mainly observed in children of preschool age and adolescents, with a mean age of52.8±40.0months. Patients one-year-old or younger had the highest infection rate; with increased age, the rate of MP infection gradually reduced, and approximately 80% of MP infections occurred in children seven years old or younger. However, RSV mainly infected infants, especially children six months old or younger. The RSV-infected patients had a mean age of 4.3±7.9months. Children 6.5 months old or younger accounted for 80% of the total RSV infections, and nearly 95% RSV infections occurred in children 10.5 months old or younger.

**Table 1:** Correlation analysis between meteorological factors and MP/RSV infection in Hangzhou.

Daily meteorological data	RSV		MP	
	R	*P*	R	*P*
Male/female	1.8:1		1.3:1	
Age (months)	4.3±7.9		52.8±40.0	
Minimum temperature (°C)	-0.804	<0.001	0.370	<0.001
Maximum temperature (°C)	-0.699	<0.001	0.329	<0.001
Average temperature (°C)	-0.772	<0.001	0.359	<0.001
Temperature variance (°C)	0.175	0.001	-0.066	0.208
Relative humidity (%)	-0.137	0.009	0.018	0.727
Average rainfall (mm)	-0.138	0.008	-0.012	0.820

Temperature variance refers to the daily temperature difference.

### Seasonality and meteorological factors

**Fig 2:**
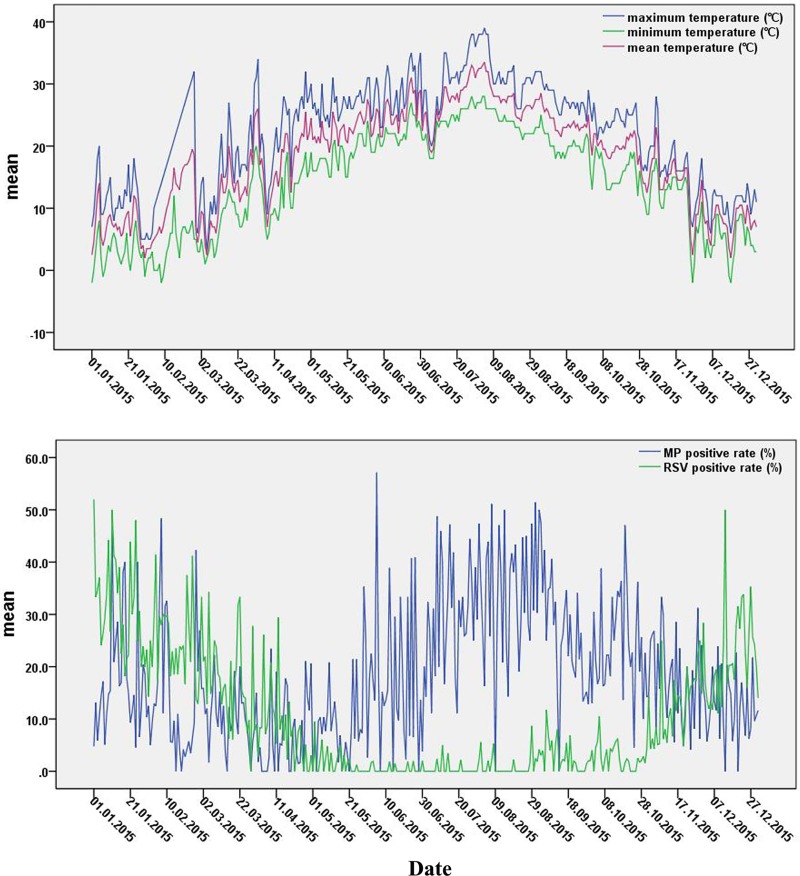
Trends in the MP/RSV infection rate by temperature variations and date.

The meteorological data from Hangzhou in 2015indicated that the lowest temperature was -2°C, the highest temperature was 39°C, the average temperature was 18.1°C, the average relative humidity was 73.8% and the average rainfall was 4mm (Table 2). The seasonal distributions of the infection rates for MP and RSV were different. The seasonal trends for MP suggested that infection occurred throughout the year, with a single peak between June and October and a positive detection rate of25.0% during that period. That was significantly higher than the MP infection rates of 14.6% in winter and 8.9% in spring. RSV infection was predominantly prevalent in winter, and the infection rate was 26.1%. However, the RSV infection rate was less than 5.0% between May and October, as shown in Fig 2.

**Table 2:** Descriptive statistics for RSV and MP infection and meteorological data between January 2015 and December 2015 in Hangzhou.

Daily meteorological data	Mean	Standard deviation	Minimum	Percentile			Maximum
				25th	50^th^	75th	
Minimum temperature (°C)	14.1	8.1	-2.0	6.5	15.0	21.0	28.0
Maximum temperature (°C)	22.1	8.4	3.0	15.0	24.0	29.0	39.0
Average temperature (°C)	18.1	8.0	2.0	11.3	19.5	24.5	33.5
Temperature variance (°C)	8.0	3.9	1.0	6.0	8.0	10.0	27.0
Relative humidity (%)	73.8	14.5	28.0	64.0	75.0	87.0	97.0
Average rainfall (mm)	4.0	9.0	0.0	0.0	0.0	4.0	86.0
Daily RSV positive rate (%)	10.4	12.0	0.0	0.0	5.1	18.2	52.0
Daily MP positive rate (%)	18.4	12.9	4.8	8.5	16.0	26.4	57.1

Temperature variance refers to the daily temperature difference.

### The relationship between the infection rate of MP/RSV and the meteorological factors

**Fig 3:**
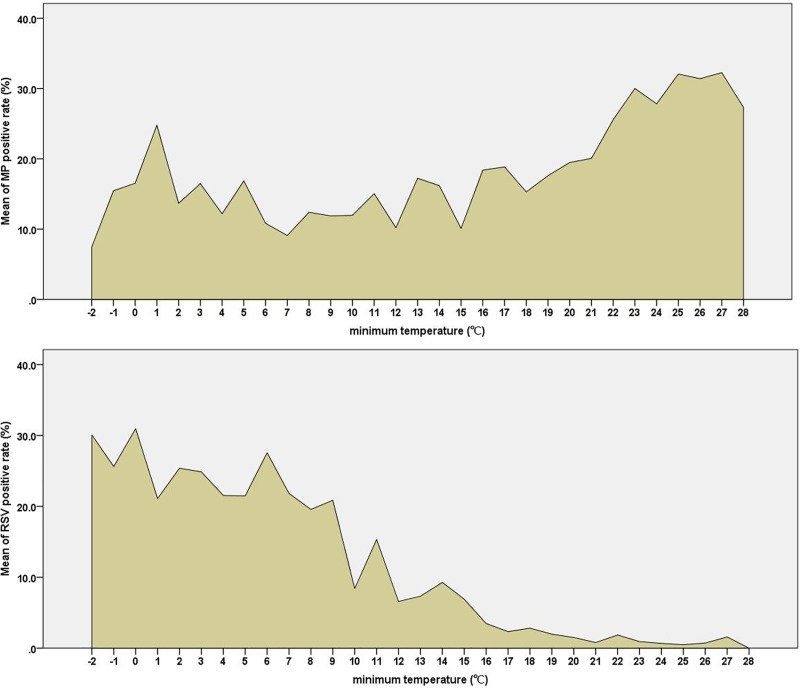
Trends in the MP/RSV infection rate among children by minimum monthly temperature in Hangzhou, 2015.

According to the results of correlation analysis, there was a positive correlation between minimum temperature, maximum temperature, average temperature, and the MP infection rate (*P* < 0.001), with the lowest temperatures having the strongest correlation. There was no significant correlation with temperature variance, relative humidity and average rainfall (*P*> 0.05), as shown in Table 1. Moreover, the RSV infection rate was negatively associated with temperature, with stronger correlations at the lowest temperatures (r = -0.804, *P*<0.001), and negatively associated with relative humidity and average rainfall (r = -0.137, *P*<0.01 and r = -0.138, *P*<0.01),as shown in Table 1.When the lowest temperature was more than 20℃, the detection rate of MP was gradually increase with the increase of the lowest temperature. When thelowesttemperature was less than9°C, the RSV infection rate was more than 20%, and when it was higher than 9°C, the RSV infection rate sharply decreased with the increase of the temperature (Fig 3).

### The lag effect and the cumulative effect of temperature on MP/RSV infection

**Fig 4:**
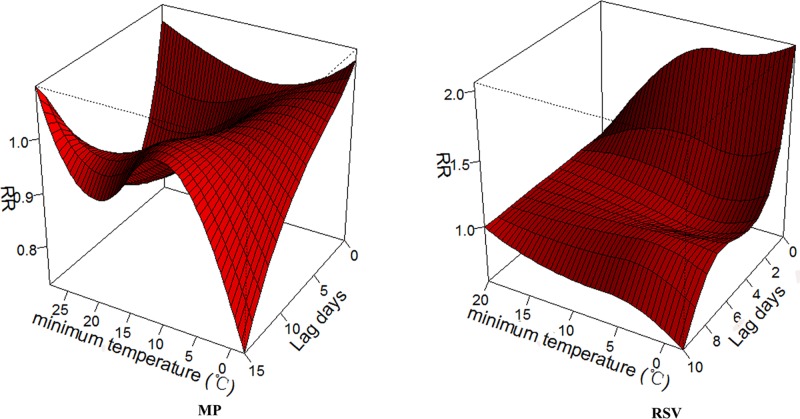
Lag associations and dose effect between temperature (baseline: 20°C) and MP and RSV infection rates using the DLNM model and controlling for holiday effects, relative humidity, day of the week and long-term trends. Temperature had evident lag on the RSV infection rate and MP infection rate.

**Fig 5:**
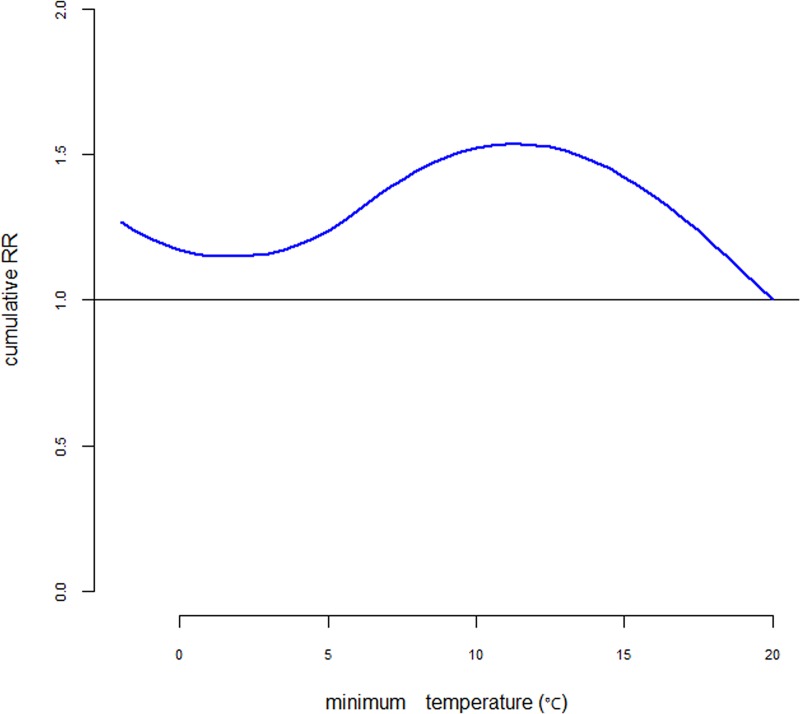
Cumulative relative risks of temperature on the RSV infection rateusing the DLNM model. Generally, the maximum impact of temperature variation on the detection of RSV occurs at lag two day, the maximum Relative Risk (RR) was approximately 1.5. The highest RR of RSV infection occurred at lag 1–2 days after the temperature decrease, and the RR increased gradually with the temperature decrease.

Although temperature was not an independent risk factor for MP or RSV infection, temperature had an important influence on infection rates. We found that temperature had evident lag and cumulative effects on the RSV infection rate. Temperature also had an evident lag effect on the MP infection rate; however, there was no evident cumulative effect between temperature and MP infection (Fig 4). Generally, the maximum impact of temperature variation on the detection of RSV occurs at lag two day, the maximum Relative Risk (RR) was approximately 1.5. The highest RR of RSV infection occurred at lag 1–2 days after the temperature decrease, and the RR increased gradually with the temperature decrease (Fig 5)

## Discussion

In this study, 36500 paediatric pneumonia cases in the Hangzhou area were used to investigate pneumonia aetiology. The results suggested MP and RSV were the main pathogens responsible for pneumonia and that infection rates were higher in males than in females. Based on the characteristics of these two pneumonia epidemics, infants up to six months of age were a high-risk group for RSV infection, suggesting that these infants cannot obtain RSV protective antibodies from their mother and that their immune system is immature. Preschool children were a high-risk group for MP infection, which probably relates to the spread of MP infection mainly through the respiratory tract; because preschool children are often crowded in densely populated places, such as schools, this may increase the chances of disease transmission.

Some studies report that MP and RSV infection have geographic land seasonal differences predominantly because climatic conditions (temperature, relative humidity and rainfall) have changed [10–12].It is evident that there are seasonal differences in the prevalence of respiratory tract infection in Hangzhou, especially related to temperature. Thus, it is necessary to investigate whether the infection rates for these pathogens are associated with meteorological factors in Hangzhou, China.

Our data suggest that MP infection positively correlated with temperature. Summer and autumn were the highest-incidence seasons for MP infection, and the infection rate was 25% when the minimum temperature was more than 20°C.Additionally, the infection rate of MP gradually increased with the increase in the minimum temperature. Because the optimum growth temperature of MP is 36–37°C and summer and autumn are the hottest months of the year, these higher temperature conditions are of benefit to MP replication. Conversely, our study suggested the RSV infection was negatively associated with temperature, especially low temperature. Low temperatures have also been found to favour RSV survival in southeast China [13], Nepal [14], Brazil [15], and Malaysia [16]. In our study, RSV infection was predominantly prevalent in winter, with an infection rate of 26.1%. This may be because winter is the coldest season of the year in Hangzhou, and its average temperature was below 10°C. When the minimum temperature was higher than 9°C, the RSV infection rate was greater than 20%. Furthermore, the temperature showed obvious lag and cumulative effects on RSV infection. On the one hand, infants and young children have immature immune systems, which may be further suppressed in the winter, so they may be more susceptible to respiratory infection. On the other hand, RSV is highly resistant to cold and remains stable in low-temperature environments, which make it is easier to spread. In addition, low temperatures promote respiratory tract spasms and ischaemia due to capillary contraction in children, resulting in weakened ciliary movement and hence weakened removal of RSV in the respiratory epithelium. All these factors favour the multiplication of RSV.

An association between detection rates and relative humidity has been reported in Germany [17], Hong Kong, Singapore, and Vancouver [18]. In this study, MP infection had no obvious correlation with relative humidity and rainfall, and the RSV infection was negatively associated with relative humidity and rainfall. The underlying reasons for the effects of humidity and rainfall on RSV infection are unclear; perhaps dry conditions favour the transmission of RSV, and rainy days could decrease outdoor activities and increase close contact with children with previously acquired RSV infection.

## Conclusions

The variation in infection rates for multiple respiratory pathogens may be caused by the differences in countries, geographical positions, meteorological factors and the diagnostic methods used. The epidemiology of MP and RSV were different, and they had some correlation with climate factors. These data suggest that temperature especially plays an important role in their prevalence. Thus, clinicians should take appropriate measures to protect high-risk groups during high-incidence seasons. The timely and effective detection of these certain pathogens may help guide clinical treatment to reduce mortality in children with pneumonia.

## Abbreviations

MP: mycoplasma pneumoniae
RSV: respiratory syncytial virus
RT-PCR: real-time polymerase chain reaction
DIF: direct immunofluorescence
DLNM: distributed lag non-linear model.
